# Pyrimidine Derivatives as Selective COX-2 Inhibitors with Anti-Inflammatory and Antioxidant Properties

**DOI:** 10.3390/ijms252011011

**Published:** 2024-10-13

**Authors:** Beata Tylińska, Anna Janicka-Kłos, Tomasz Gębarowski, Paulina Nowotarska, Stanisława Plińska, Benita Wiatrak

**Affiliations:** 1 Department of Organic Chemistry, Wroclaw Medical University, Borowska 211A, 50-556 Wroclaw, Poland; 2 Department of Basic Chemical Sciences, Wroclaw Medical University, Borowska 211a, 50-556 Wrocław, Poland; stanislawa.plinska@umw.edu.pl; 3 Department of Biostructure and Animal Physiology, The Wroclaw University of Environmental and Life Sciences, Kożuchowska 1/3, 51-631 Wroclaw, Poland; tomasz.gebarowski@upwr.edu.pl (T.G.); paulina.nowotarska@upwr.edu.pl (P.N.); 4 Department of Pharmacology, Wroclaw Medical University, Mikulicza-Radeckiego 2, 50-345 Wrocław, Poland; benita.wiatrak@umw.edu.pl

**Keywords:** cancer therapy, inflammation, COX inhibition

## Abstract

Pyrimidine derivatives exhibit a wide range of biological activities, including anti-inflammatory properties. The aim of this study was to investigate the effects of tested pyrimidine derivatives on the activity of cyclooxygenase isoenzymes (COX-1 and COX-2), antioxidant properties, and their ability to inhibit the growth of inflammatory cells. In vitro tests were conducted to assess the ability of pyrimidine derivatives L1–L4 to inhibit COX-1 and COX-2 activity using the TMPD oxidation assay (N,N,N′,N′-tetramethyl-p-phenylenediamine). The compounds’ ability to inhibit the growth of lipopolysaccharide (LPS)-stimulated THP-1 (human leukemia monocytic) monocyte cells and their impact on reactive oxygen species (ROS) levels in an inflammatory model were also evaluated. The binding properties of human serum albumin (HSA) were assessed using UV–Vis spectroscopy, circular dichroism (CD), and isothermal titration calorimetry (ITC). Among the tested pyrimidine derivatives, L1 and L2 showed high selectivity towards COX-2, outperforming piroxicam and achieving results comparable to meloxicam. In the sulforhodamine B (SRB) assay, L1 and L2 demonstrated dose-dependent inhibition of LPS-stimulated THP-1 cell growth. Additionally, ROS assays indicated that these compounds reduced free radical levels, confirming their antioxidant properties. Binding studies with albumin revealed that L1 and L2 formed stable complexes with HSA. These results suggest that these compounds could serve as a basis for further research into anti-inflammatory and anticancer drugs with reduced toxicity.

## 1. Introduction

**Figure 1:**
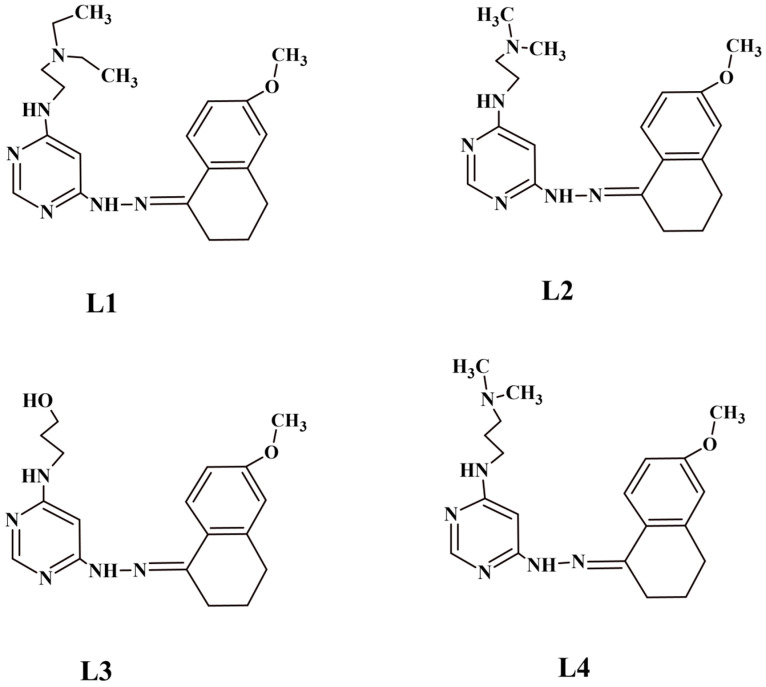
Pyrimidine derivatives (ligands).

Based on GLOBOCAN data, it is expected that by 2040, the incidence of colorectal cancer will increase to 3.2 million new cases and the number of deaths to 1.6 million [1]. In Poland, in 2021, the National Cancer Registry recorded 171,558 new cases and 93,652 deaths due to malignant tumors. The data show that the most common cancers in men were prostate, lung, and colorectal cancer, while in women, they were breast, lung, and colorectal cancer [2]. According to literature reports, cyclooxygenase (COX) is overexpressed in cancer cells [3,4]. Cyclooxygenase-1 (COX-1, described in 1971 [5]) and cyclooxygenase-2 (COX-2), described in the 1990s, are known. These enzymes mediate the bioconversion of arachidonic acid to inflammatory prostaglandins (PGs) [6]. Prostaglandins are hormone-like substances involved in autocrine and paracrine signaling. They play an important role in many physiological and pathological processes occurring in the human body [7,8]. It has been shown that COX-1 and COX-2 are genetically independent proteins, which is related to their different properties [9]. Under basal conditions, cyclooxygenase-1 levels are high in most cells, while cyclooxygenase-2 levels are low or undetectable [10]. The latest data indicate that high levels of COX-2 expression occur in pathological conditions such as inflammation and in cancer cells. Research shows the relationship between the use of nonsteroidal anti-inflammatory drugs (NSAIDs) and the development of colorectal cancer. It turned out that non-steroidal anti-inflammatory drugs (NSAIDs) and cyclooxygenase inhibitors may have a beneficial effect in preventing the development and growth of colorectal cancer [11,12,13]. Studies improving the effectiveness of anticancer drugs using COX-2 inhibitors have also been described. This combination not only improves the therapeutic effect but can also reduce toxicity and chemoresistance [14,15]. In this study, we performed biological assays to evaluate the inhibitory effects of tested pyrimidine derivatives on COX-1 and COX-2 activity, their ability to suppress lipopolysaccharide (LPS)-stimulated cell growth, reduce reactive oxygen species (ROS) levels in an inflammatory model, and their binding interaction with human serum albumin (HSA). The pyrimidine derivatives L1–L4 tested in this study are newly synthesized compounds evaluated, for the first time, for their anti-inflammatory and antioxidant properties. Compounds with a pyrimidine group exhibit a wide spectrum of biological activities, including antibacterial [16,17,18,19,20,21,22,23], antidiabetic [20], anti-HIV [24], antimalarial [25,26], anticancer [19,27,28,29,30], anti-Alzheimer’s disease [31,32,33,34], anti-inflammatory [16,17,18,35,36], and analgesic effects [18,37]. In a previous article, we described the synthesis and anticancer activity of the pyrimidine derivatives L1–L4 (Figure 1).

All pyrimidine derivatives were evaluated in vitro for antitumor activity in LoVo colorectal adenocarcinoma, LoVo/DX-resistant colorectal adenocarcinoma, MCF-7 breast cancer, A549 lung cancer, cervical cancer (HeLa), human leukemic lymphoblasts (CCRF-CEM), and human monocyte cell lines (THP-1) [30]. When designing drugs, it is important to consider the pharmacokinetics of compounds, that is, the absorption, distribution, metabolism, and elimination of drugs from the body. This is now known as LADME (L-liberation, A-absorption, D-distribution, M-metabolism, and E-elimination). When a drug is administered to a patient, the molecule, once absorbed, is unchanged in the bloodstream or binds to plasma proteins. The unbound drug is the active form, while the substance bound in a complex with a protein, such as albumin, provides a storehouse for the drug, which can be released into the bloodstream over time. Albumin is a protein with important functions in the human body. Interest in it increased after information about its role as a carrier in the treatment and diagnosis of diseases [38,39]. To study protein–ligand intercalation, we used titration calorimetry (ITC), which measures the change in heat that occurs when a ligand (Figure 1) binds to a protein (albumin). Binding behavior was also studied using circular dichroism (CD) spectroscopy (used to consider changes in the secondary structure of the protein after ligand binding) and UV–Vis absorption spectroscopy.

The main findings of this work highlight the selective COX-2 inhibition and antioxidant properties of pyrimidine derivatives L1 and L2, demonstrating their potential as anti-inflammatory agents. Future studies will aim to further explore these compounds’ applications in cancer therapy and their pharmacokinetic profiles to optimize their therapeutic efficacy and reduce side effects. Additionally, new pyrimidine-based structures will be designed based on these findings to further investigate their biological activities and enhance their anti-inflammatory and anticancer potential.

## 2. Results and Discussion

### 2.1. In Vitro Cyclooxygenase Inhibition Assay

The peroxidase activity of cyclooxygenase (COX) was assessed using a colorimetric approach. Specifically, we evaluated the oxidation of N,N,N′,N′-tetramethyl-p-phenylenediamine (TMPD), which serves as a substrate for various enzymes with peroxidase activity. The reduction of prostaglandin G2 (PGG2) to prostaglandin H2 (PGH2) occurs due to the oxidation of TMPD. This enzymatic reaction leads to a change in color, which we measured at 590 nm using a microplate reader.

The impact of meloxicam and piroxicam, as well as tested compounds, on the activity of both cyclooxygenase isoforms (COX-1 and COX-2) was assessed. The incubation time was set to 2 min, following the manufacturer’s protocol. Given the well-documented adverse effects of nonsteroidal anti-inflammatory drugs (NSAIDs) like aspirin, naproxen, and ketoprofen, which predominantly inhibit COX-1 [38,39,40,41], we aimed to compare the effects of pyrimidine derivatives with meloxicam and piroxicam. Meloxicam inhibited both isoforms but exhibited a greater affinity for COX-2. In contrast, piroxicam inhibited both COX forms to a similar degree. We determined the concentrations of all tested compounds required for 50% inhibition of COX-1 and COX-2. Subsequently, IC_50_ values were calculated, and the ratio of IC_50_ values for both cyclooxygenase isoforms allowed us to assess the selectivity of the tested structures toward COX-1 and COX-2. The complete set of IC_50_ values for both enzymes and the selectivity coefficient is presented in Table 1.

Only two tested compounds (L1 and L2) exhibited cyclooxygenase activity in the in vitro assay. Both compounds demonstrated higher affinity for the COX-2 isoform than for COX-1. Furthermore, these derivatives inhibited COX-2 similarly comparably to the reference drug, meloxicam. Simultaneously, both tested compounds significantly outperformed the reference drug piroxicam in inhibiting the COX-2 isoform. The IC_50_ values for COX-2 inhibition by the tested compounds closely resembled those of meloxicam. This observation suggests that pyrimidine derivatives could serve as a promising scaffold for potent COX-2 inhibitors. Notably, the tested compounds exhibited selective inhibition of COX-2. Regarding COX-1 activity, their performance resembled that of piroxicam but surpassed meloxicam (particularly L2, which significantly inhibited COX-1 more effectively than meloxicam). This difference may be attributed to the larger binding pocket in the active site of COX-2 compared to the COX-1 isoform [42].

### 2.2. Cyclooxygenase Molecular Docking Study

**Figure 2:**
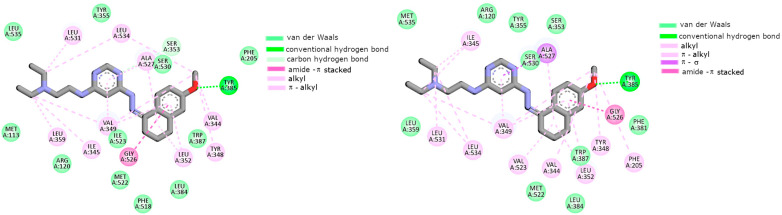
The intermolecular interactions of L1 compounds with COX-1 (top) and COX-2 (bottom).

**Figure 3:**
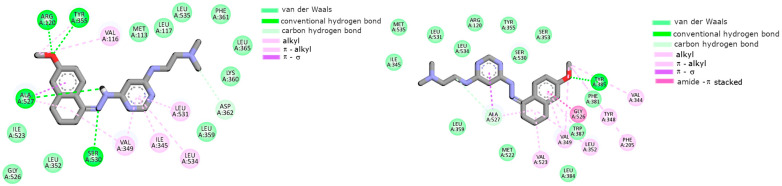
The intermolecular interactions of L2 compounds with COX-1 (**top**) and COX-2 (**bottom**).

Molecular docking was performed in order to predict the binding manner of L1 and L2 compounds to the cyclooxygenase isoforms. According to an immunoenzymatic study, both of them exhibited inhibitory activity. In our simulations, we used the crystalographic structures of COX-1 (PDB ID: 4O1Z) and COX-2 (PDB ID: 4M11) co-crystallized with the reference drug meloxicam [43]. Earlier studies showed the structural differences in the binding center of both isoenzymes. As obtained, COX-2 contains an extra binding pocket with Leu352, Ser353, Tyr355, Phe518, and Val523, which arises due to the position of Tyr355 influenced by the presence of Val523 instead of Ile523 [44,45]. Moreover, due to the replacement of His513 in COX-1 by an Arg513 amino acid residue in COX-2, additional hydrogen bonds are also possible. The obtained data are presented in Figure 2 and Figure 3. As can be seen in the Figures, both compounds could bind to the active center of the cyclooxygenases. The scoring functions, including the free enthalpy of binding, suggest that L1 and L2 can bind a little bit stronger to COX-2; however, the difference is negligible (less than 1 kcal/mol).

As can be seen, the naphthalene moiety of L1 was involved in interactions with the hydrophobic pocket created by the Phe518, Trp387, Leu352, Tyr385, Gly526, Met522, and Ala527 amino acid residues of both isoenzymes (see Figure 2). The pyrimidine ring in both cases interacted via π–alkyl interactions with two amino acid side chains, namely Val349 and Ala527. In close proximity, Ser530 was also localized, which formed L1 van der Waals interactions. The alkyl interactions with Ile345, Val349, Leu531, and Leu534 stabilized the aliphatic tail in the binding cavities of proteins. L2 exhibited a different mode of binding. The aliphatic chain of this compound was stabilized in both cases mainly by van der Waals interactions. In the binding center of COX-1, the pyrimidine ring formed π–alkyl interactions with Ile345, Val349, Leu531, and Leu534. The naphthalene ring, in this case, was able to form two hydrogen bonds with Arg120 and Tyr355. As presented in Figure 3 (bottom), the pyrimidine ring interacted with COX-2 exclusively via van der Waals forces. The exception was alanine, which formed with pyrimidine π–σ interactions. The naphthalene ring was involved in many types of interactions. For example, these were amide-π stacking interactions with Gly526 or hydrogen bonding with Tyr385.

### 2.3. Evaluation of Viability in Inflammatory Model

The monocytic cell line (THP-1) is a cell line used as a model of inflammation after preincubation with PMA [46,47,48]. To evaluate the impact of the tested compounds on the anti-inflammatory effect, we measured the total cell protein using the SRB assay. After differentiation with PMA, THP-1 cells treated with LPS induce inflammatory cytokines through Toll-like receptors (TLRs). TLRs are pattern recognition receptors that defend against bacterial infection, and in our model, this was induced by lipopolysaccharide (LPS). The lipopolysaccharide from the bacterial cell wall we used was a TLR4 ligand [47].

**Figure 4:**
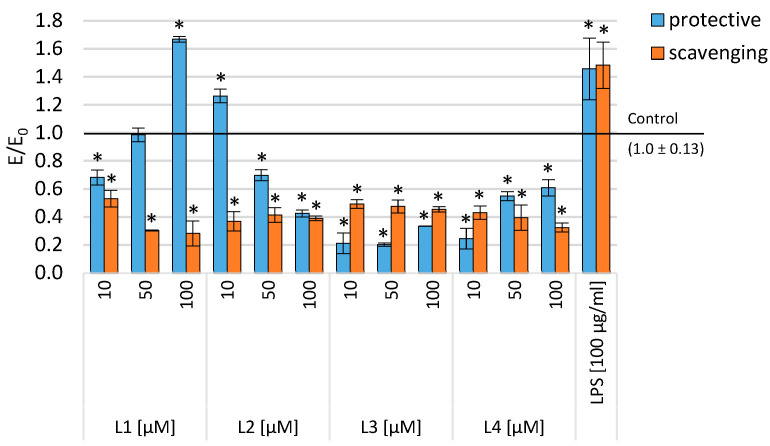
Cell viability (SRB assay) after incubation with the tested compounds in various concentrations in THP-1 cells. Data are presented as means and SD. * *p* < 0.05—significant difference from the negative control.

To assess the effect of different doses of the tested compounds on cell growth, we treated THP-1 macrophages with 10, 50, and 100 µM of the tested compounds. At higher doses of the tested compounds, a dose-dependent inhibition of THP-1 macrophage growth was observed (Figure 4). During a 48 h incubation of the monocytic cell line (THP-1) preincubated with PMA and treated with pyrimidine derivatives and then with 100 µg/mL of LPS (to assess the protective effect against the intensification of inflammation), a statistically significant reduction in inflammation (measured by the viability of inflammatory cells) was observed for L3 and L4 across the entire concentration range, and for the compound L2, in the range of 50–100 µM and L1 for concentrations of 10 and 50 µM. Moreover, in the case of the L1 compound, inflammation persisted at the highest concentration tested, and in the case of the L2 compound, a reduction in inflammation was observed compared to the positive control (incubation with LPS only), but a statistically significantly greater inflammation was observed compared to the control (THP-1 preincubated with PMA only). In the context of combating inflammation, initial incubation with 100 µg/mL of LPS followed by treatment with the tested compounds resulted in a statistically significant reduction in the number of inflammatory cells across the entire concentration range of the tested compounds compared to the negative control (Figure 4). Cells treated with the tested compounds at a concentration of 50–100 µM showed a 30–60% growth inhibition compared to controls after 48 h, regardless of the tested system (protective against the intensity of inflammation or treatment of inflammation).

### 2.4. Level of Intracellular ROS

**Figure 5:**
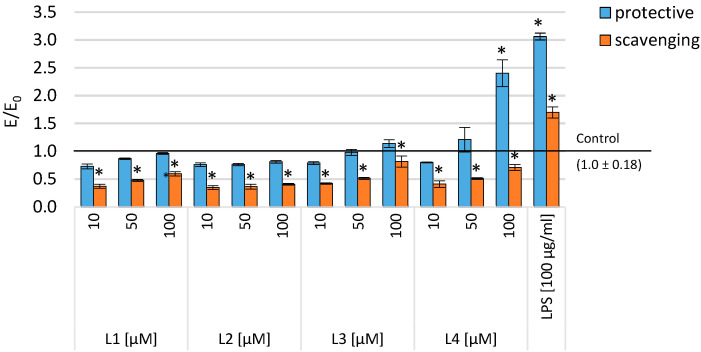
ROS measured with DCF-DA assay after incubation with the tested compounds in various concentrations in THP-1 cells. Data are presented as means and SD. * *p* < 0.05—significant difference compared to the negative control.

Reactive oxygen species (ROS) are generated as a result of cellular metabolic processes. An increase in ROS levels, leading to oxidative stress, can be caused by various factors such as hypoxia or inflammation [49]. Numerous studies indicate that ROS plays a significant role as a mediator of pro-inflammatory processes, and oxidative stress and inflammation are closely interconnected. Cyclooxygenases (COX-1 and COX-2) can influence ROS levels. Simultaneously, an elevation in oxygen concentration and free radicals may induce increased activity of these enzymes [25,26,27]. In our experiments, we employed the DCF-DA assay to assess ROS levels in response to induced oxidative stress using 100 µg/mL of lipopolysaccharide (LPS). The results of this test are presented in Figure 3. In studies concerning protective properties against oxidative stress, a model of inflammation based on THP-1 cells (preincubated with PMA) was employed. Subsequently, the cells were treated with the tested compounds and exposed to 100 µg/mL of lipopolysaccharide (LPS) for 1 h. As a result of these experiments, protective properties against oxidative stress were observed for all investigated compounds compared to the control group treated with LPS alone. Furthermore, when compared to the culture incubated in a medium without LPS, the level of reactive oxygen species (ROS) was similar to the control for compounds L1 and L2 across the entire concentration range. The other compounds exhibited a similar ROS level only at the lowest tested concentration. Additionally, in the context of scavenging oxygen free radicals, after 24 h of incubation with LPS (100 µg/mL), a statistically significant reduction in free radical levels was observed, both compared to the culture treated with LPS alone and to the control group (Figure 5).

### 2.5. Determination of Binding Constants

**Figure 6:**
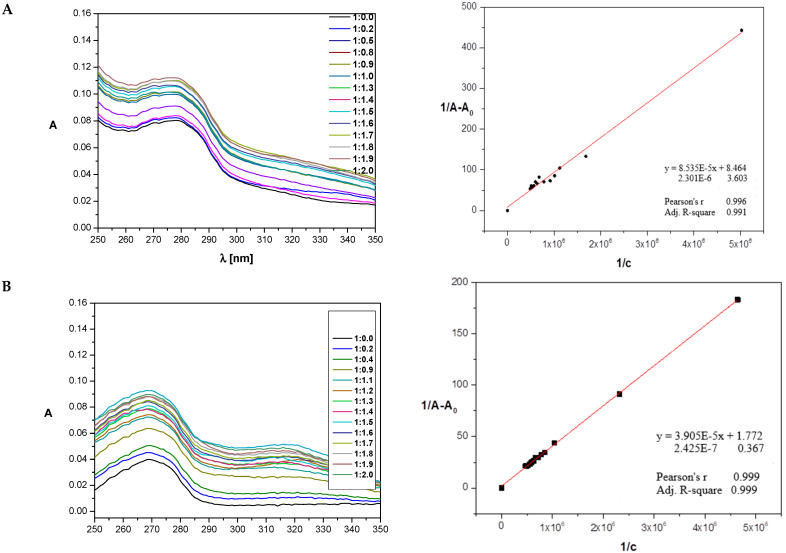

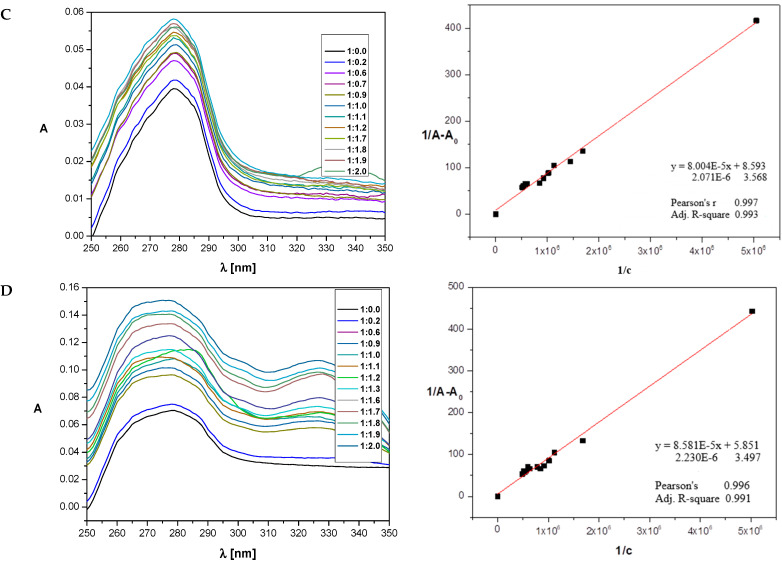
The UV absorption spectra and the Benesi–Hildebrand data analysis result plot for titration of HSA by ligands L1 (**A**), L2 (**B**), L3 (**C**), and L4 (**D**).

To check the pharmaceutical potential of studied compounds, the interactions between pyrimidine derivatives and human serum albumin were studied. The fluorescence quenching study spectra, UV–Vis, and circular dichroism spectra after adding the tested compound to the protein probe were measured. Due to the overlapping effect in fluorescence characteristics, it was impossible to determine the nature of the interactions between HSA and the studied ligands. During the fluorescence experiment, UV–Vis spectra of the studied systems were detected. These experiments were used for the determination of stability constants for the HSA–ligand complexes. The addition of the ligand to the protein solution increased the absorbance observed for all studied compounds. Based on that fact, and to obtain binding constants, the Benesi–Hildebrand procedure was performed (Figure 6 and Table 2) [50,51].

Results from the UV study show that in all studied systems, HSA formed stable complexes with L1, L2, and L3 and with comparable affinity, while the complex with L4 was onefold weaker, which was also confirmed by CD studies. The free energy was almost identical for HSA–L1 and HSA–L3 and close to the HSA–L4 value, while for HSA–L2, it was the lowest.

**Figure 7:**
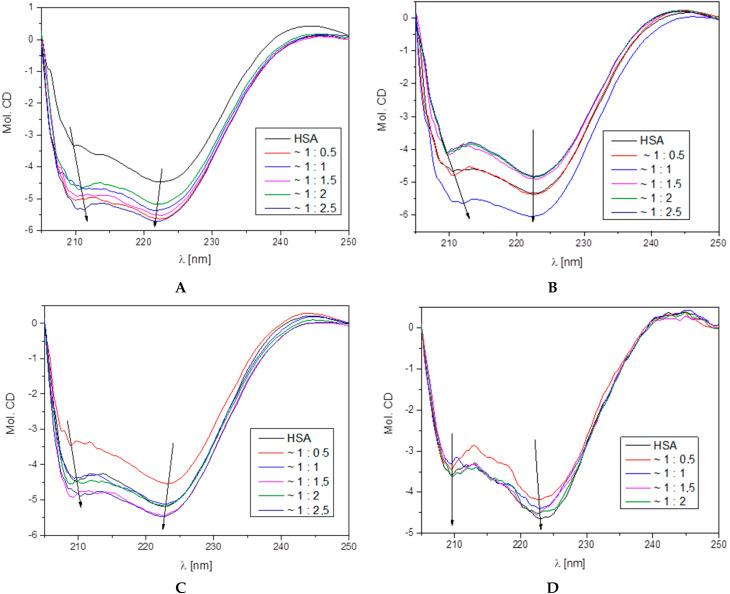
Far UV-CD spectra of human serum albumin in the presence of the ligands L1 (**A**), L2 (**B**), L3 (**C**), and L4 (**D**). pH 7.4, T = 25 °C.

Human serum albumin is very sensitive to interactions with other ligands, which is observed in the changes in circular dichroism spectra [52]. HSA has a characteristic pattern on the CD spectrum-characteristic bands, negative peaks near 209 and 220 nm for α-helix and one negative band around 215 nm for the β-sheet structures [53]. The measured CD spectra for proteins in the absence and presence of all tested compounds are summarized in Figure 7, while the obtained results based on the experimental spectra using the CD Multivariate SSE program are presented in Table 3. The addition of the studied compounds into the HSA solution slightly changed its spectra. On the HSA spectra, two negative α-helix bands, near 210 nm and 222 nm, were present. The observed changes during the addition of successive portions of the tested compounds were not very significant. The biggest reduction in the percentage of α-helix was observed for L1 (around 4%), then for L3 (around 3%) and for L2 and L4 (around 2% and 1.5%). Similarly, for β-sheet structure. Loss of α-helix percentage resulted in an increase in β-sheet content. Analyzing all the changes in the protein structure, it can be deduced that all the tested compounds did not destabilize the secondary structure of the protein.

**Figure 8:**
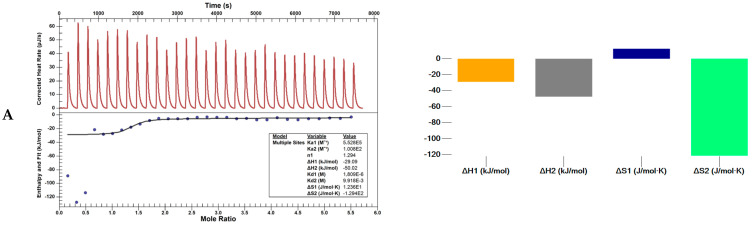

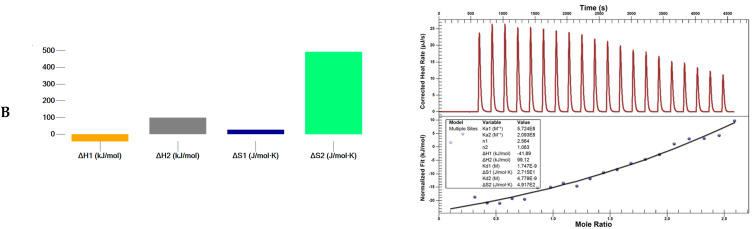
The best-fit ITC data for the titration of 3 mM L1 (**A**) and L4 (**B**) into 0.014 mM HSA in HEPES buffer, pH 7.4 and T = 25 °C.

The binding features of pyrimidine derivatives compounds L1 and L4 with HSA at a physiological pH were investigated by ITC experiments to gain insights into the energetics of the complexation processes (Figure 8). Determination of the entropic and enthalpic contributions to the standard Gibbs energy made it possible to determine in detail the nature of the interactions between the studied ligands and the human albumin protein.

Calorimetric measurements indicate that the HSA bound L1 and L4 in an exothermic process (ΔH < 0) with a positive change in the entropy. All thermodynamic parameters obtained for HSA–L1 and HSA–L4 systems are collected in Table 4. Usually, when the multiple binding sites on a macromolecule are present and are independent, an independent model is used. For studied systems referring to the shape of the obtained isotherm, it can be assumed that the binding sites are not identical. The best fitting for the obtained results is for the multiple-sites model, which may be related to the existence of non-identical binding sites or the interaction between them [54]. The values of the first enthalpies of binding were negative, while the binding of L4 at the second site required the supply of energy to the system (ΔH has a positive value). A different trend could also be seen in the determined number of moles at both binding sites for both studied systems. The increase in entropy values in HSA–L4 indicates that the system becomes more disordered despite the positive enthalpy contribution to the binding process.

The value of binding constant results obtained by spectroscopic and calorimetric methods differed. However, the results for L4 differed regardless of the method, which could explain the different behavior of L4 in biological studies. Regardless of the method used to determine the Gibbs free energy, it was negative (Table 2 and Table 4) and of a similar order in all cases, which indicates that in all of the studied processes, the binding process was spontaneous.

## 3. Materials and Methods

### 3.1. Cyclooxygenase Inhibition Assay

The assay kit provided by the manufacturer (Cayman Chemical, Ann Arbor, MI, USA) included 150 μL of assay buffer, 10 μL of heme, and 10 μL of either COX-1 or COX-2. We prepared all samples in triplicate, adding 10–250 μM of the analyzed compounds along with 10 μM of methanol, ethanol, and dimethyl sulfoxide (DMSO). Subsequently, we added 20 μL of TMPD to all wells. Finally, arachidonic acid was introduced to activate the reaction, which lasted for 2 min. After this incubation period, we determined the extent of TMPD oxidation using the Multiscan Go microplate reader with 590 nm (Thermo Fisher Scientific, Waltham, MA, USA).

### 3.2. Molecular Modeling

The structures of L1 and L2 were optimized using density functional theory (B3LYP/6-31G(d, p)) in the Gaussian 09 package [55,56,57]. The calculations were performed by using a polarizable continuum model (PCM), taking into account the water solution [58,59].

Molecular docking studies were performed using the AutoDock4.2 package, and a standard protocol was followed to predict the binding mode and the free energy of binding [60]. We used as an input a specially prepared chain A of cyclooxygenases. The polar hydrogens, Gasteiger charges, and solvent parameters were calculated using a program, and the water molecules and all co-factors from the crystallographic structures were removed. The binding sites were defined using a grid of 60 × 60 × 60 points with a grid space of 0.375 Å. The center of the box was located in the binding site according to the meloxicam coordination. The docking parameters were the same as in previous works on this topic [61,62].

The validation of docking was performed by the docking of meloxicam into the crystal structures of cyclooxygenases and comparing its position in the original crystallographic structure. The obtained results were visualized using Biovia Discovery Studio Visualizer.

### 3.3. Biological Evaluation

#### 3.3.1. Cell Line and Culture Conditions

The monocyte cell line THP-1, obtained from the American Type Culture Collection (ATCC, Manassas, VA, USA), served as a common inflammation model. THP-1 cells were cultured in RPMI-1640 medium supplemented with 10% fetal bovine serum (FBS), 2 mM of L-glutamine, and 25 μg/mL of gentamicin (Biological Industries, Beit Haemek, Israel). Incubation conditions included 5% CO_2_, 37 °C, and 95% humidity. Cells were evaluated twice a week, and if confluence exceeded 70%, they were used in biological assays or subcultured. For all experiments, cells were seeded at a density of 25,000 cells/well and incubated for 24 h to regenerate in a CO_2_ incubator.

#### 3.3.2. Tested Compounds

The tested compounds were dissolved in DMSO (Sigma-Aldrich, Darmstadt, Germany) to a final concentration of 10 μM and stored at −20 °C. Before use, the compound solution was kept at room temperature until thawing, and then samples with three different concentrations (100 µM, 50 µM, and 10 µM) were prepared for each compound. The concentrations of the tested compounds were prepared in a culture medium. At the highest concentration of each compound, the DMSO content did not exceed 1%.

#### 3.3.3. Experimental Design

To generate THP-1 macrophages, THP-1 monocytes were treated with 5 ng/mL of PMA (phorbol 12-myristate 13-acetate) (Sigma-Aldrich, Darmstadt, Germany) for 48 h and incubated at 37 °C with 5% CO_2_ [46]. PMA acts as a differentiation factor, converting nonadherent monocytic THP-1 cells into adherent macrophages. After 48 h of PMA treatment, investigated compounds at concentrations of 10–100 µM or LPS (lipopolysaccharide) (Sigma-Aldrich, Darmstadt, Germany) at a concentration of 100 µg/mL were added to the culture. Cells were incubated with these compounds for an additional 24 h. After the appropriate time, the solution was removed and re-treated, again with investigated compounds or LPS, for 24 h (for the SRB assay) or 1 h (for the DCF-DA assay).

#### 3.3.4. SRB Assay

Viability evaluation was conducted using the SRB dye (sulforhodamine B), which binds to cellular proteins and provides information about protein content. After 24 h of cell regeneration, the SRB assay was performed. In this assay, we also performed a DMSO (0.1%) control. One culture plate served as a control. It was fixed with a cold TCA (trichloroacetic acid) solution at a final concentration of 10% *w*/*v* for 1 h at 4–8 °C. The remaining culture plates were incubated with the tested compounds for 48 h at 37 °C in a 5% CO_2_, 95% humidity environment. After the incubation period, all plates were fixed with a cold TCA solution. Plates were washed four times with running water and air-dried at room temperature (RT). A 0.4% SRB solution in 1% *v*/*v* acetic acid was added to the plates for 30 min. The excess stain was rinsed off five times with a 1% acetic acid solution. The SRB dye bound to intracellular proteins was dissolved using 10 mM of Trizma base for 30 min with stirring on a shaker. Finally, the absorbance was measured at 540 nm using a Multiscan Go microplate reader. All reagents used in the study were purchased Sigma-Aldrich (Darmstadt, Germany)

#### 3.3.5. DCF-DA Assay

DCF-DA (2′,7′-dichlorofluorescein diacetate) is a fluorescent dye used to measure free radical levels. Once it diffuses into cells, DCF-DA is deacetylated by esterases, converting it into a nonfluorescent compound. In the presence of reactive oxygen species (ROS), this compound is oxidized to form 2′,7′-dichlorofluorescein (DCF), which emits fluorescence. The DCF-DA solution was prepared by dissolving 1 mg of DCF-DA in 2.05 mL of 100% ethanol. The solution was diluted in sterile filtered water (BioReagent, suitable for cell culture, W3500, Sigma Aldrich, Darmstadt, Germany) to achieve a final concentration of 10 μM. The intracellular free radical levels were assessed under conditions with exogenous stress. Cells were exposed to 100 μM of H_2_O_2_ (hydrogen peroxide), an ROS generator. We used a fluorescence control (cells without DCF-DA solution). To measure ROS levels, the DCF-DA solution was incubated with the cells for an additional hour, and fluorescence was read using a microplate reader (λ_ex_ = 485 nm, λ_em_ = 535 nm).

#### 3.3.6. Statistical Analysis

In the conducted bioassays, the normality of distribution was assessed using the Shapiro–Wilk test and the Levene test was employed to verify equal variance. A one-way ANOVA with Tukey’s post hoc test was also applied. All analyses were carried out using Statistica 13.1 software. A significance level of (*p* < 0.05) was assumed for all tests. Based on the conducted tests, the statistical power was calculated to be greater than 80%. To assess COX inhibition, mathematical models were developed using the GraphPad PRISM 8.0.2 software, on the basis of which the IC_50_ was determined. IC_50_ values were estimated based on a four-parameter Hill slope logistic model. IC_50_ is the concentration that inhibits cyclooxygenase activity by 50%. The determined IC_50_ values are summarized in Table 1.

### 3.4. Spectroscopic Studies

#### 3.4.1. UV–Vis Spectroscopy

UV–Vis spectra were reordered in the Jasco V750 spectrophotometer (Jasco, Tokyo, Japan) with automatic baseline correction. All spectra for the 1 × 10^−6^ M HSA solution in the absence and presence of studied compounds were measured in a pH 7.4 and HEPES buffer as a solvent at 25 °C. Tested compounds were dissolved in DMSO, and buffer was added so the final concentration of DMSO in the measured samples was less than 1%. The measurements were carried out in quartz cells with a 10 mm path length in the 298 K with a 0.5 nm interval. Each spectrum is the average of 3 accumulations. The solution of HSA was titrated with small amounts of the studied 1 × 10^−4^ M L1, L2, L3, and L4 ligands. Changes in absorption peak after ligand addition were used to calculate the apparent association constants K_a_ by the Benesi–Hilderbrand (B–H) method [50].

#### 3.4.2. Circular Dichroism

Circular dichroism (CD) spectra were measured on the Jasco J-1500 magnetic circular dichroism spectrometer (JASCO International CO, Tokyo, Japan). Tested compounds were dissolved in DMSO, and buffer was added so the final concentration of DMSO in measured samples was less than 1%. All spectra for the 1 × 10^−6^ M HSA solution in the absence and presence of 1 × 10^−6^ M of the studied compounds were measured in pH 7.4 and HEPES buffer as a solvent with baseline correction in 25 °C. All spectra were measured in the range of 205–260 nm at a scan rate speed of 50 nm/min, with a response time of 1 s and 0.5 cm path length (for L1, L2, and L3) and 0.1 cm (L4). Ligands were partially added into 3 mL of protein solution. Experiments were performed to obtain the appropriate protein-to-compound molar ratios equal to 1:0.0, 1:0.5, 1:1.0, 1:1.5, 1:2.0, and 1:2.5. The analysis of the obtained results was in the CD Multivariate Calibration Creation and CD Multivariate SSE programs (JASCO International CO, Tokyo, Japan) with PCR method. Protein concentrations were converted for mean residue molar concentrations.

#### 3.4.3. Nano ITC Calorimeter

ITC experiments were carried out using a Nano ITC calorimeter (TA Instruments, New Castle, DE, USA) with a standard volume of 1.0 mL at 25 °C. All the solutions were prepared in deionized water (>18 Ω) and HEPES buffer solution. Tested compounds were dissolved in DMSO, and buffer was added so the final concentration of DMSO in measured samples was less than 1%. All the solutions used to fill both the cell and the syringe were degassed 15 min before analysis. The reference cell was filled with deionized water. The HSA solution was an analyte (0.14 mM) in the cell, and the studied compounds (3 mM) were as a titrant in the syringe. Each time, a freshly prepared solution of titrant was taken up in a 250 μL injection syringe and titrated into protein solution. A solution of the studied compounds in a different number of injections was added after the calorimeter finalized the primary equilibration. The interval between the injections was 200 s for L1 and L4, leaving 200 s at the beginning of the experiment without injection. The stirring rate was set at 300 rpm in each experiment. The control experiments to determine the heat of the dilution of each compound were performed by injecting the same concentration of ligand into the buffer. The calorimeter was operated using Nano ITC Run software (www.tainstruments.com). The enthalpy change for each injection was calculated by integrating the area under the peaks. All the data obtained were analyzed using the NanoAnalyze v. 3.1.2 program provided by the manufacturer.

An ‘independent’ and ‘multiple-sites model’ were used to evaluate the results. ITC data was collected using independent measurements, and reproducible data was employed.

The enthalpy change (∆H), entropy change (∆S), association constant (Ka), and binding stoichiometry (n) were obtained by the least-squares minimization process and taken as the best-fit values. ΔG was calculated from the Gibbs Helmholtz equation (ΔG = −RTlnK_a_ = ΔH − TΔS).

## 4. Conclusions

Studies on pyrimidine derivatives have demonstrated their significant potential in inhibiting cyclooxygenase isoenzyme activity, particularly COX-2, which is crucial in anti-inflammatory and anticancer therapies. Compounds L1 and L2 exhibited greater selectivity towards COX-2 than COX-1, making them promising candidates for COX-2 inhibitors. Additionally, these compounds showed antioxidant capabilities by reducing ROS levels in the THP-1 inflammatory cell model. Their binding to albumin confirmed the stability of these complexes, which is key to their bioavailability and pharmacological action. These results highlight the potential of pyrimidine derivatives as anti-inflammatory drugs with selective action and low toxicity.

## Figures and Tables

**Table 1:** Half maximal inhibitory concentration, IC_50_ values (mean ± SD; n = 3) calculated for COX-1 and COX-2 enzymes after 2 min with the tested compounds, COX selectivity ration; N/C–not calculable (based on the concentrations tested); t-test for the evaluation of COX-1 and COX-2 activity compared to the control compound piroxicam (^ *p* < 0.05) and the control compound meloxicam (* *p* < 0.05).

Compounds	Cyclooxygenase Inhibition Assay IC_50_ [µM]		COX Selectivity Ratio IC_50(COX-2)_/IC_50(COX-1)_
	COX-1	COX-2	
L1	105.8 ± 22.39	74.6 ± 3.03 ^^^	0.70
L2	91.0 ± 3.40 *	76.8 ± 1.20 ^^^	0.84
L3	N/C	N/C	-
L4	N/C	N/C	-
Piroxicam	87.4 ± 5.10	80.1 ± 1.54	0.92
Meloxicam	128.8 ± 5.78	76.4 ± 7.91	0.59

**Table 2:** The association constant (Ka) of HSA–Ligand complexes.

Ligand	L1	L1	L3	L4
Ka [dm^3^/mol]	9.92 × 10^4^	4.54 × 10^5^	1.07 × 10^5^	6.82 × 10^4^
∆G [J/mol]	−2.85 × 10^4^	−3.23 × 10^5^	−2.87 × 10^4^	−2.76 × 10^4^

**Table 3:** The percentage of content of the secondary structure elements in HSA solution in the absence and presence of studied compounds. Calculations were performed in the CD Multivariate SSE program based on experimental spectra.

**HSA–L1** **Molar Ratio**	**% α-Helix**	**% β-Sheet**	**% β-Turn**	**% Other**
1:0.0	69.4	5.8	8.5	16.3
1:0.5	66.7	10.1	8.2	15.0
1:1.0	65.8	9.0	8.5	16.7
1:1.5	66.2	12.2	8.0	13.6
1:2.0	65.6	10.7	8.3	15.4
1:2.5	65.5	11.9	8.1	14.5
**HSA–L2** **Molar Ratio**	**% α-Helix**	**% β-Sheet**	**% β-Turn**	**% Other**
1:0.0	68.2	8.6	8.2	15.0
1:0.5	68.6	7,3	8.3	15.8
1:1.0	67.7	6.2	8.6	17.5
1:1.5	68.2	11.7	7.7	12.4
1:2.0	67.1	7.3	8.5	17.1
1:2.5	66.5	7.7	8.6	17.2
**HSA–L3** **Molar Ratio**	**% α-Helix**	**% β-Sheet**	**% β-Turn**	**% Other**
1:0.0	69.6	5.5	8.4	16.5
1:0.5	64.6	6.9	9.0	19.5
1:1.0	64.9	9.8	8.5	16.8
1:1.5	65.0	10.0	8.4	16.6
1:2.0	67.1	9.4	8.7	14.8
1:2.5	66.5	9.6	8.6	14.3
**HSA–L4** **Molar Ratio**	**% α-Helix**	**% β-Sheet**	**% β-Turn**	**% Other**
1:0.0	65.4	3.5	8.9	22.2
1:0.5	55.5	7.5	9.7	27.3
1:1.0	66.2	5.2	9,1	19.5
1:1.5	60.2	9.0	8.9	21.9
1:2.0	64.2	6.4	9.1	20.3

**Table 4:** Thermodynamic parameters obtained for L1–HSA and L4–HSA systems in ITC experiments. Data for the titration of 3 mM L1, L4 into 0.14 mM HSA in HEPES buffer, pH 7.4 and T = 25 °C.

		K_a_ (M^−1^)	n	ΔH (kJ/mol)	K_d1_ (M)	ΔS (J/mol·K)	ΔG (J/mol)
L1	1	5.528 × 10^5^	1.29	−29.1	1.809 × 10^−6^	1.236 × 10^1^	−3.278 × 10^4^
	2	1.008 × 10^2^	3.61	−50.1	9.918 × 10^−3^	−1.294 × 10^2^	−1.154 × 10^4^
L4	1	5.724 × 10^8^	2.56	−41.9	1.747 × 10^−9^	2.715 × 10^1^	−4.999 × 10^4^
	2	2.093 × 10^8^	1.06	99.1	4.779 × 10^−9^	4.917 × 10^2^	−4.742 × 10^4^

## Data Availability

Raw data are available after contacting the corresponding authors.
